# Acquired methemoglobinemia in infancy secondary to diarrhea: a case report

**DOI:** 10.11604/pamj.2024.49.17.43733

**Published:** 2024-09-19

**Authors:** Mariam Hany Aly, Hessa Mohammed Bukhari, Mohammed A.H. Aldirawi, Lemis Yavuz

**Affiliations:** 1 Mohamed Bin Rashid University, Dubai Health, Dubai, United Arab Emirates,; 2 Al Jalila Children's Hospital, Dubai Health, Dubai, United Arab Emirates

**Keywords:** Methemoglobinemia, gastroenteritis, infant, central venous sinus thrombosis, case report

## Abstract

Methemoglobinemia (MetHb) is a life-threatening condition that reduces the oxygen-carrying ability of hemoglobin. Acquired methemoglobinemia usually results from exposure to specific oxidizing agents. Symptoms and complications depend on the MetHb level, which can sometimes be fatal. We present a case of a 6-week-old infant exhibiting hypoxia alongside gastroenteritis, fever, poor oral intake and low activity, with confirmed methemoglobinemia in blood gas analysis. Despite negative results in the workup for underlying causes, including genetic testing conducted later, methylene blue administration only partially reduced methemoglobinemia level. Treatment involved managing diarrhea by transitioning to a hydrolyzed formula. Interestingly, an incidental discovery of partial central venous sinus thrombosis occurred during the diagnostic process, although no established correlation with methemoglobinemia was evident in the literature. This case report illustrates the complex presentation of methemoglobinemia in a previously healthy infant, occurring concurrently with gastrointestinal infection and unexpected thrombosis. It underscores the need for interdisciplinary collaboration and comprehensive management in addressing such multifaceted clinical scenarios in pediatric practice. This case emphasizes the importance of considering diarrhea as a possible cause of methemoglobinemia, especially in infancy. It also highlights the need for increased clinical awareness and prompt management approaches towards the various presentations of acquired methemoglobinemia in pediatric populations.

## Introduction

Methemoglobinemia is a potentially life-threatening etiology of cyanosis necessitating heightened clinical attention due to its potentially fatal effects [1]. It is characterized by a reduction in the oxygen-carrying capacity of circulating hemoglobin. This occurs due to the conversion of the iron species from the reduced ferrous [Fe^2+^] state to the oxidized ferric [Fe^3+^] state. At this stage, the ferric iron cannot effectively bind and transport oxygen. Elevated levels of methemoglobin lead to functional anemia [1]. Methemoglobinemia can have either a congenital or acquired origin. In congenital forms, deficiencies in cytochrome b5 reductase or abnormal hemoglobin, specifically hemoglobin M, result in the incapacity for effective reduction [2]. Acquired methemoglobinemia may be due to exposure to direct oxidizing agents like benzocaine or indirect oxidation like nitrates [3]. Indirect oxidation by nitrites can be caused by exposure to exogenous toxins such as that of the bacteria *Escherichia coli (E. coli)* and *Campylobacter* [4,5]. These microorganisms induce methemoglobinemia by introducing a substantial nitrite burden. This excess nitrite subsequently oxidizes the ferrous iron in hemoglobin to a ferric state, creating methemoglobin. [4,5].

This case report details a 6-week-old infant presenting with both methemoglobinemia and gastroenteritis. The methylene blue only partially reduced methemoglobin levels. Diarrhea control with a hydrolyzed formula was the treatment. This case highlights the significance of considering diarrhea as a potential trigger for methemoglobinemia in infants and emphasizes the importance of prompt management and clinical vigilance in addressing acquired methemoglobinemia in pediatric patients.

## Patient and observation

**Patient information:** a previously healthy 6-week-old baby boy presented to our hospital with a 1-day history of fever, vomiting, and diarrhea. The fever ranged between 37.8°C and 38.3°C. He experienced two episodes of non-bloody, non-bilious vomiting and five episodes of diarrhea. Additionally, he showed reduced oral intake and low activity. The infant had no prior medical history and was born to healthy, non-consanguineous parents. His antenatal and birth history were unremarkable, and he had received the birth vaccine. The child had been growing and developing normally until the onset of illness. There was no family history of genetic or hematological disorders.

**Clinical findings:** upon presentation, the infant appeared dusky in color. His vitals revealed an oxygen saturation of 86%, tachycardia, tachypnea, and a delayed capillary refill time of 2-3 seconds.

**Diagnostic assessment:** initial blood gas analysis indicated metabolic acidosis (pH: 7.21, partial pressure of carbon dioxide (PCO_2+_): 27.8 mmHg, Bicarbonate (HCO_3_): 13.9 mmol/L, Carboxyhemoglobin: 9.3 g/dL, Lactate: 2.1mmol/L), and methemoglobin levels were highly suggestive of methemoglobinemia (FMetHb: 63.8%). A fluid bolus of 20 mL/kg normal saline over 20 minutes was administered, and a repeat blood gas showed a marked worsening in metabolic acidosis (pH: 7.053, PCO_2_: 30.9 mmHg, HCO_3_: 8.9 mmol/L, FMetHb: 64.5%, Lactate: 8.1mmol/L). Another bolus of 20 mL/kg normal saline was given (Table 1).

**Table 1:** diagnostic investigations conducted during hospital encounter

Investigation	Results	Reference range
Hemoglobin	9.6 g/dL	11.5-16.5 g/dL
Platelets	708 103/uL	210-500 103/uL
White blood cells	34.9 103/uL	5.0 - 19.0 103/uL
Absolute lymphocytic count	19.20 103/uL	3.00-16.00 103/uL
C-Reactive protein	23.2 mg/L	0-5 mg/L
Procalcitonin	0.33 ng/mL	<0.5 ng/mL
Bicarbonate	8.0 mmol/L	15-26 mmol/L
Bilirubin, total	1.27 mg/dL	0-1.2 mg/dL
Albumin	2.9 g/dL	3.8 -5.4 g/dL
**Respiratory screening panel (nasopharyngeal PCR )**		
Influenza A	Negative	
Influenza B	Negative	
Human metapneumovirus A + B	Negative	
Adenovirus	Negative	
Respiratory screening panel multiplex	Negative	
**Gastrointestinal panel**		
Enteroaggregative *Escherichia coli*	Positive	
**Blood film analysis**		
Blood film shows severe leukocytosis, mild microcytic hypochromic anemia and leukoerythroblastic blood picture indicating bone marrow stress. Sepsis? hemolysis?		
**Chest x-ray**		
No significant abnormality noted		

**Diagnosis:** methemoglobinemia associated with gastroenteritis.

**Figure 1:**
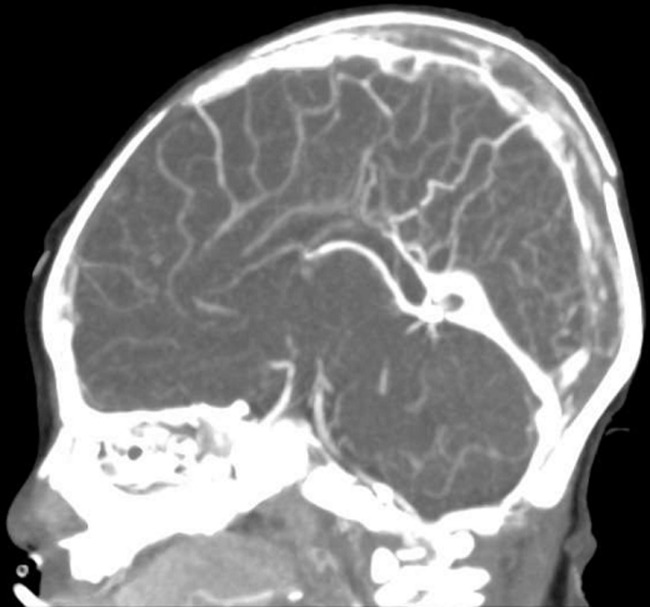
sagittal maximum intensity projection (MIP) showing a thrombus in the superior sagittal sinus

**Figure 2:**
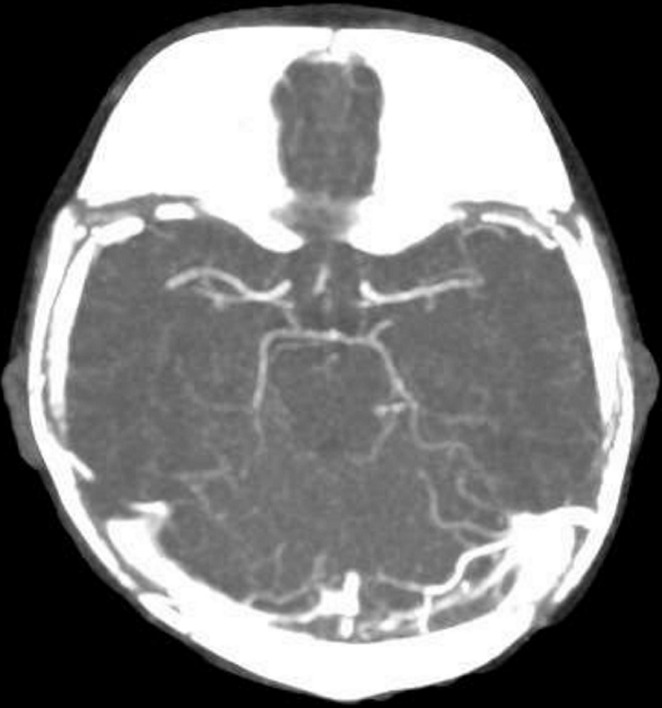
axial maximum intensity projection (MIP) showing a thrombus in the left transverse sinus

**Therapeutic interventions:** in the emergency department, given the worsening oxygen saturation, the infant was placed on a two-liter nasal cannula of oxygen. There was no improvement in oxygen saturation, so he was placed on BiPAP (Bilevel positive airway pressure) (pressure: 14/5 cmH20, rate: 30 beats per minute, FiO_2_: 100%). The infant was then transferred to the pediatric intensive care unit (PICU) for further management. Methylene blue (2 mg/kg) was administered. Repeat capillary blood gas after 6 hours showed a MetHb of 2.2 (pH: 7.26, PaCO_2_: 27.4, Bicarb: 13.9, Lactate: 2, MetHb: 2%). In the next 48 hours, the patient had hypokalaemia K 2.6, and blood sugar was borderline around 60 mg/dl. However, he remained stable on intravenous fluid and oxygen 1.5 L nasal cannula. The MetHb level fluctuated up to (15%) but didn't need a second dose of methylene blue. Given the very early onset of methemoglobinemia and the absence of predisposing factors, the genetic, metabolic, and hematology teams were consulted. Rapid genetic testing for methemoglobinemia was initiated. The echocardiogram revealed a small atrial communication from left to right, mostly patent foramen ovale, but otherwise normal cardiac anatomy. The patient was later shifted to the ward on day three of admission and was irritable. The repeated hemoglobin level was 7.1 g/dl, and the gastrointestinal panel showed Enteroaggregative *Escherichia coli (E. coli)*. He received a packed red blood cell transfusion. Also, a cranial ultrasound (US) was done, which revealed partial superior sinus thrombosis. A subsequent CT scan confirmed partial central venous sinus thrombosis involving the superior sagittal and left transverse sinus (Figure 1, Figure 2). Ophthalmology screening was reassuring. The workup for thrombosis was negative, and it did not require treatment.

Over one week and while waiting for blood test results, the baby continued to have watery diarrhea 3-5 times per day, hypoalbuminemia reached 2.4, and the methemoglobin level was fluctuating. Additionally, he was not gaining weight. However, he did not need more than oxygen supplements and intravenous fluid. During this time, the baby was on a regular baby formula. Given the negative testing results for methemoglobinemia causes, including genetic, the working diagnosis was methemoglobinemia secondary to gastroenteritis. Hence, the baby was placed on a hydrolyzed formula. In the next 72 hours, the diarrhea ceased, and weight gradually increased. The following comprehensive metabolic panel indicated normal methemoglobin and albumin levels.

**Follow-up and outcome of interventions:** follow-up in the clinic over the next three months revealed normal weight gain and normal methemoglobin level.

**Patient perspective:** the parents expressed satisfaction with the healthcare provided, understanding that the cause of their baby's presentation was likely related to gastrointestinal infection rather than genetic factors. They were provided with instructions on recognizing warning signs necessitating a visit to the emergency department, including persisting fever, vomiting, diarrhea, episodes of bluish discoloration, or any other concerning complaints.

**Informed consent:** the parents were briefed on the intention behind publishing the case report and provided consent for the inclusion of their baby's clinical data and imaging in the journal publication.

## Discussion

Methemoglobinemia is a functional anemia. It decreases hemoglobin's ability to carry oxygen, which occurs when methemoglobin exceeds 1% in the bloodstream. This decrease happens because some or all of the iron atoms in hemoglobin shift from a ferrous [Fe^2+^] state to an oxidized ferric [Fe^3+^] state. The iron cannot efficiently bind and transport oxygen in this oxidized state [1]. The pathophysiology of this condition remains unidentified. Several contributing factors play a role in its manifestation. Infants' erythrocytes exhibit heightened susceptibility to methemoglobin formation, primarily due to fetal hemoglobin, which is more prone to oxidation than adult hemoglobin [6].

Signs and symptoms of methemoglobinemia mimic other illnesses, including respiratory, cardiac, and central nervous system illnesses, shock, or even death [2]. The suspicion of methemoglobinemia should arise in instances of unexplained cyanosis and hypoxemia. Like our patient, his saturation did not improve despite oxygen being provided. Significant methemoglobinemia symptoms are linked to MetHb levels. When the level exceeds 70%, it can cause death. Methemoglobinemia could be primary or secondary, so it is crucial to differentiate acquired cases that are more common than inherited ones. The most common cause is exposure to oxidizing agents [7]. Interestingly, gastroenteritis in infancy has been identified as a possible cause of methemoglobinemia and was reported in a few cases [4]. The data suggested that altered gastrointestinal flora due to infections and higher intestinal flora in infants promote the proliferation of intestinal flora, which, in turn, potentiates the conversion of nitrates to nitrites from dietary intake [8]. The diagnosis of methemoglobinemia is typically confirmed through analysis of arterial or venous blood gas using co-oximetry [2].

Management depends on methemoglobinemia level and clinical manifestations. However, it is crucial to identify and treat the trigger factor to correct the level. Observation is enough in asymptomatic patients. Primary treatment involves 1% methylene blue with an initial 1-2 mg/kg dose. Ascorbic acid can be adjunctive. Non-responders may need blood exchange or hyperbaric oxygen therapy [2]. Similarly, changing to a hydrolyzed formula and controlling the diarrhea were the treatments in our case.

An interesting finding in the case was the unexpected discovery of partial superior sinus thrombosis coexisting with methemoglobinemia. Despite an extensive review of the existing literature, no definitive correlation between thrombosis and methemoglobinemia was found. The absence of a clear relationship between the two conditions raises questions about whether there is a shared pathophysiological mechanism between the two or if it is just a rare co-occurrence. Given the critical nature of methemoglobinemia and thrombosis, future studies should explore the possible connections and implications for patient management.

The importance of this case report lies in the multifactorial presentation of methemoglobinemia in a previously healthy 6-week-old infant. The diagnosis of methemoglobinemia in the presence of a gastrointestinal infection with *E. coli* and the unexpected finding of partial superior sinus thrombosis show a complex diagnostic and therapeutic scenario not commonly encountered in pediatric practice. It shows the need for multidisciplinary investigation and intervention to effectively address the primary pathology and associated complications.

## Conclusion

Methemoglobinemia is a life-threatening condition and should be considered in unexplained hypoxemia. The potential causes could be as severe as congenital or simple as gastroenteritis. Treatment depends on symptoms and level, and it is essential to treat the underlying condition.
